# Prenatal modulation of NADPH-oxidase reverses the deranged GABA switch and rescues behavioral deficits in valproate ASD rat model

**DOI:** 10.3389/fphar.2025.1571008

**Published:** 2025-05-30

**Authors:** Basma A. Yasseen, Hadeer Abdelkhalek, Sara Gohar, Yasmin Hatem, Hajar El-Sayed, Aya A. Elkhodiry, Aya Galal, Aya Samir, Nouran Al-Shehaby, Malak W. Elbenhawi, Rehab Hamdy, Christine S. Prince, Azza G. Kamel, Mohamed A. Badawy, Ghada F. Soliman, Soha Aly ElMorsy, Tamer M. Gamal El-Din, Ebtehal El-Demerdash, Sameh S. Ali, Engy A. Abdel-Rahman

**Affiliations:** ^1^ Tumor Biology Research Program, Research Department, Children’s Cancer Hospital Egypt, Cairo, Egypt; ^2^ Cancer Research UK Scotland Institute, Glasgow, United Kingdom; ^3^ Preclinical and Translational Research Center, Faculty of Pharmacy, Ain Shams University, Cairo, Egypt; ^4^ Department of Biotechnology, Faculty of Science, Cairo University, Cairo, Egypt; ^5^ Department of Medical Pharmacology, Faculty of Medicine, Cairo University, Cairo, Egypt; ^6^ Department of Pharmacology, University of Washington, Seattle, WA, United States; ^7^ Department of Pharmacology, Faculty of Pharmacy, Ain-Shams University, Cairo, Egypt; ^8^ Department of Pharmacology, Faculty of Medicine, Assiut University, Assiut, Egypt

**Keywords:** ASD, GABA D/H switch, synaptosomes, KCC2, NOX, shikonin

## Abstract

**Indroduction:**

Impaired depolarizing-to-hyperpolarizing (D/H) switch of gamma-aminobutyric acid (GABA) is reported during brain development in rodent valproate-model of autism spectrum disorder (VPA-ASD). We hypothesize that this impairment triggers NADPH oxidases (NOXs)-induced reactive oxygen species (ROS) overproduction.

**Methods:**

Here, we followed the impact of prenatal exposure to VPA on the synaptic protein expression of potassium chloride cotransporter 2 (KCC2), sodium potassium chloride cotransporter 1 (NKCC1) and, in brains of male and female Wistar rats during infantile (P15), juvenile (P30) and adult (P60) stages. We also assessed alterations in synaptic NOX isoforms 2 and 4 (NOX2 and NOX4) activities and expressions in developing rat brains.

**Results:**

Our findings revealed a significant reduction in KCC2 expression and a concomitant increase in NOX activity and NOX4 expression in synaptosomes of VPA-exposed rats, particularly at P15 and P30. Prenatal exposure to shikonin, (10 mg/kg/day, intraperitoneal (i.p.) into pregnant dam, daily from G12.5 until birth), ameliorated these effects by reducing synaptic protein expression of NOX4, generally quenched synaptic NOX activity and enhanced synaptic protein expression of KCC2. Indeed, shikonin reversed VPA-induced sociability deficits in ASD rats.

**Discussion:**

These results suggest that targeting the NOX-ROS pathway may be a potential therapeutic strategy for ASD.

## Graphical Abstract

**Figure:**
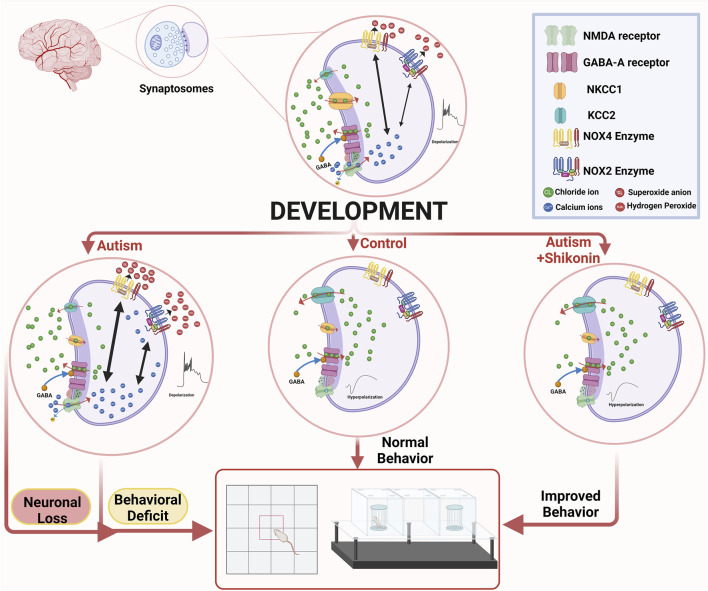
Suppression of GABA D/H shift in mature ASD brain, leads to sustained activation of N-methyl-D-aspartate (NMDA) -type glutamate receptors, that in turn triggers reactive oxygen species (ROS)-mediated neuronal death. Shikonin restores GABA D/H Switch, modulates NOX4 in VPA-synaptosomes, and ameliorates behavior deficits in VPA-ASD rats. The graph was created in BioRender. Lab, P. (2024) BioRender.com/k03q771.

## Highlights

• Impaired GABA D/H switch was observed in VPA-ASD rat synaptosomes.
• Impaired GABA D/H switch was linked to increased NOX4 activity and expression in juvenile VPA-ASD rat synaptosomes.
• Prenatal shikonin exposure prevents disruptions in GABA D/H switch and modulates NOX4 in VPA synaptosomes.
• Prenatal exposure to shikonin ameliorates behavioral deficits in VPA-ASD rats.

## 1 Introduction

ASD has been highlighted as a multifaceted neurodevelopmental behavioral disorder, characterized by a life-long impairment in social interaction and the existence of restricted repetitive behaviors (Hodges et al., 2020). The global prevalence of ASD has increased to 1 in every 100 children (Zeidan et al., 2022). Although evidence supports the hereditary transmission of autism, studies indicated that exposure to some environmental factors during critical periods of development is associated with heightened susceptibility to ASD (Gardener et al., 2009). One such environmental risk factor associated with ASD is the prenatal exposure to some anti-seizure medications, including VPA (Brumback et al., 2018; Chaliha et al., 2020). Clinical studies have shown that maternal consumption of VPA during pregnancy is linked to a higher risk of ASD and intellectual disability (Bjørk et al., 2022; Hernández-Díaz et al., 2024). Similarly, rodents that were exposed to VPA during the prenatal stage, exhibit behavioral impairments, that closely resemble deficits observed in children diagnosed with ASD (Chaliha et al., 2020; Wang et al., 2018).

In the developing brain, excitatory and inhibitory synaptic connections are expansively formed between thousands of differentiating neurons (Zhao et al., 2022). The establishment of a finely tuned balance between synaptic excitation and inhibition plays a fundamental role in shaping neural circuits (Leonzino et al., 2016). Accordingly, the imbalance between excitatory and inhibitory neural transmission within brain synapses and hence the term “Synaptopathy” has been implicated in several forms of cognitive deficits, including social behavioral deficits associated with ASD (Won et al., 2013). The primary factors dictating this equilibrium are glutamate and GABA neurotransmitters, which, respectively generate the principal excitatory and inhibitory inputs in the postsynaptic neurons. At early stages of development, however, stimulation of GABAARs leads to membrane depolarization, resulting in excitation (Ben‐Ari et al., 1989). The depolarizing action of GABA, at early postnatal life is ostensibly induced by the higher expression of a chloride importer NKCC1, while the expression of chloride exporter KCC2 is being minimal in immature brains. This in turn results in the accumulation of chloride in immature neurons and, upon opening of GABAARs, efflux of this anion promotes membrane depolarization. Later in development, GABAergic neurotransmission shifts from being depolarizing to be hyperpolarizing, due to the development-associated upregulation of the chloride exporter KCC2, leading to lower intracellular chloride levels that drive influx of chloride upon stimulation of GABAARs (Dzhala et al., 2005; (Rivera et al., 1999; Yamada et al., 2004). In rodents, the GABA D/H switch is instigated shortly after delivery and is accomplished, by the completion of the initial postnatal week (Valeeva et al., 2013). Previous studies documented deficits in the developmentally associated GABA D/H switch in rodent models of ASD, resulting in sustained GABA-mediated excitation (Haratizadeh et al., 2023; Tyzio et al., 2014). In this context, studies reported that *in utero* VPA-induced ASD and Fragile X syndrome (FXS) ASD models, both of which exhibited an abnormal GABA D/H shift during birth that persists in juvenile animals. This shift was characterized by downregulation of KCC2 in hippocampi of infantile and juvenile VPA rats as well as FXS mice, resulting in increased intracellular chloride levels (Haratizadeh et al., 2023; Tyzio et al., 2014). A comparable reduction in the expression of KCC2 was also documented in the cortices of adult VPA mice as well as in postmortem brain tissues obtained from adult individuals diagnosed with ASD (Savardi et al., 2020). Downregulation of KCC2 has also been observed in primary neurons isolated from murine Lgdel+/−, which is a model for DiGeorge Syndrome, an infrequent ASD stemming from the deletion of a specific segment on chromosome 22 (Amin et al., 2017).

In the developing brain, GABAergic hyperpolarization triggers the expression of glutamate excessive neurons. Additionally, GABA-mediated depolarization alleviates the magnesium (Mg^2+)^ blockade, and thus activates NMDA-type glutamate receptors with a subsequent rise in the influx of intracellular calcium ions and cell depolarization. Accordingly, the developing brain exhibits heightened activity primarily due to the synergistic effects of depolarizing GABA and the substantial, prolonged NMDA-mediated excitatory postsynaptic currents (EPSCs). This catalyzes synaptic plasticity, alongside the production of oscillatory patterns that regulate the activity-dependent formation of functional neural units. Indeed, the absence of the developmentally associated GABA D/H switch results in sustained activation of NMDA-type glutamate receptors and starts a cascade of excitotoxicity, that eventually induces neuronal loss (reviewed in: Ben-Ari, 2014). In fact, pathological activation of NMDA receptors (NMDARs), incites excitotoxicity through the enduring generation of superoxide anion radical (O_2_^∙−^), which promotes oxidative stress and neuronal death (reviewed in: Armada-Moreira et al., 2020). Alternatively, perturbations in ROS homeostasis in neuronal cells instigate downstream signaling cascades that could culminate in further NMDA receptor stimulation in a vicious cycle (Nguyen et al., 2011). Notably, studies utilizing both pharmacological and genetic manipulations indicate that the O_2_^∙−^ generated by the activation of NMDARs predominantly derives from NOXs (Brennan et al., 2009; Han et al., 2016; Kovac et al., 2014; Wang and Swanson, 2020). NOXs are crucial source of ROS in the brain and produce O_2_^∙−^ as their main function. NOXs comprises several isoforms NOX 1–5, DUOX1, and DUOX2. The primary product of NOX enzymes is believed to be the formation of O_2_^∙−^, which spontaneously dismutates into H_2_O_2_ through superoxide dismutase (SOD). Current evidence, however, suggests that various NOX isoforms, such as NOX4, DUOX1, and DUOX2, are primarily generating H_2_O_2_. This observed increase in H_2_O_2_ levels could be linked to the swift dismutation of O_2_^∙−^ (reviewed in: Nayernia et al., 2014). The fact that NOXs are plasma membrane-localized, rationalizes the release of O_2_^∙−^ extracellularly and cell-to-cell dissemination of excitotoxic damage that has been documented both *in vitro* and *in vivo* (Wang and Swanson, 2020). We have previously shown that NOX2 and NOX4 isoforms, localize to synaptic sites and that they serve as main contributors to ROS generation at the synapses (Abdel-Rahman et al., 2016; Khalifa et al., 2017).

In ASD, glutamate-mediated excitotoxicity has been identified as a significant contributor of oxidative stress (Essa et al., 2013; Nisar et al., 2022). The susceptibility of synapses to stressful insults encountered in neurological disorders is remarkably heightened owing to high levels of ROS generation in synapses, alongside their high-energy demands (Mattson and Liu, 2002). Here, we propose a new vicious cycle involved in ASD behavioral deficits that include impaired development-associated GABA D/H shift, glutamate excitotoxicity and NOX overactivation at synapses. The output of the cycle is an enduring generation of synaptic ROS. The precise dynamics of NOX2 and NOX4 within ASD-developing brain synapses remains incompletely comprehended. Moreover, synaptic protein expression of GABA D/H switch machinery within ASD developing brain is not resolved yet. Here, we used spin-trapping electron paramagnetic resonance (EPR) spectroscopy to compare NOX-dependent O_2_^∙−^ production in synaptosomes isolated from brains of VPA-induced ASD rats at infantile (P15), juvenile (P30) and adult (P60) stages. We also monitored the developmental changes in NOX- induced oxygen consumption as well as H_2_O_2_ generation in ASD synaptosomes using the Oroboros O2k Station equipped with a fluorescence detection module. The differential protein expression of NOX2, NOX4 subunits, KCC2 and NKCC1, during development were also determined in ASD synaptosomes. We also explored if intervening with this deleterious ROS cycle through NOXs inhibition could prevent impairment in the development-associated D/H shift of synaptic GABA and subsequent behavioral deficits in VPA-induced ASD rats. To this end, we evaluated the influence of modulation of NOXs through prenatal exposure to shikonin on synaptic protein expression of KCC2 and NKCC1, in VPA synaptosomes. Moreover, the effect of prenatal exposure to shikonin in ameliorating behavioral deficits in VPA-induced ASD rats was also explored.

## 2 Methods

### 2.1 Animals

Male and female wistar rats were purchased from the Research Institute of Medical Entomology (Giza, Egypt), and kept in pathogen-free, ventilated cages in 12-h light/12-h dark cycles at constant temperature 24°C and 50% relative humidity, with free access to food and water in UResearch Animal Facility (URAF) and Preclinical and Translational Research Center, School of Pharmacy at Ain Shams University animal facility. All animal care and experimental procedures were conducted in adherence to the guidelines by the ARRIVE (Animal Research: Reporting of *InVivo* Experiments) https://arriveguidelines.org/. Animal care and experimental protocols were approved by the URAF-IACUC Institutional Animal Care and Use committee (Approval No.17/22).

### 2.2 Establishment of the VPA-induced rodent model of ASD

**FIGURE 1:**
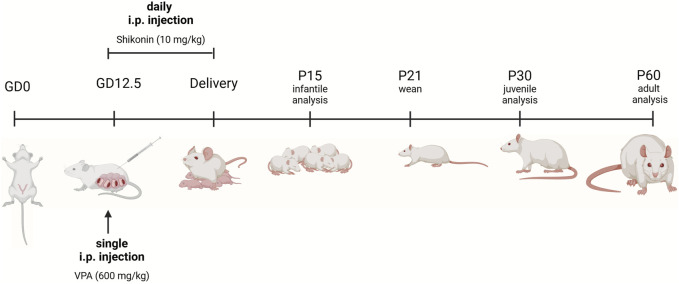
Experimental timeline. For VPA-treated pregnant dams, a single intraperitoneal (i.p.) injection of VPA in sterile 0.9% NaCl) (600 mg/kg) was given on gestational day 12.5 (GD 12.5). Pregnant dams treated with shikonin received a single dose of VPA (600 mg/kg, IP) GD 12.5, in addition to daily i.p. injection of 10 mg/kg of shikonin starting from GD 12.5 until delivery. Sample collection and litter management. Control at dams gave birth and cared for their offspring under standard conditions until weaning (P21), with no additional experimental treatments. Pups were euthanized, and their brains were collected for analysis at different developmental stages. Analysis time points included P15 (infantile), P30 (juvenile), and P60 (adult). Rats prenatally exposed to shikonin were sacrificed and brains were analyzed at P60. The figure was created in BioRender. Lab, P. (2024) BioRender.com/y94l407.

VPA rat model of ASD is generated as previously described (Tartaglione et al., 2019). Female wistar rats were mated, and the first pregnancy day known as (gestational day 0, G0) was verified by the detection of a vaginal plug. On gestational day 12.5, pregnant rats received a single i.p. injection of 600 mg/kg sodium valproate (NA VPA) (Sigma-Aldrich, P4543) in 0.9% (w/v) sodium chloride. Dams were housed individually with their own litters that were weaned on postnatal day 21 (P21). After weaning, rats were segregated by sex and housed in separate cages. Experiments were performed on either sex on: postnatal days (P) 15, 30, 60 unless stated otherwise. For prenatal exposure to shikonin, i.p. administration of 10 mg/kg/day, of shikonin (Sigma-Aldrich, S7576); to pregnant dam was performed daily from G12.5 until birth Figure 1. The selected dose was based on a previously published study. (Zhu et al., 2023).

### 2.3 Isolation of synaptosomes

Isolation of synaptosomes was conducted as described elsewhere (Abdel-Rahman et al., 2016; Khalifa et al., 2017). Briefly, forebrains were expeditiously extracted, dissected and homogenized utilizing a dounce homogenizer in ice-cold isolation buffer (0.32 mol/L sucrose, 1 mmol/L EDTA, 10 mmol/L Tris-HCl buffer with a pH of 7.4, and 10 mmol/L glucose). The homogenate was centrifuged, and the resulting supernatant was collected, while the pellet was reconstituted in half the volume of isolation buffer, homogenized once more, and subjected to a repeat centrifugation. The resultant supernatants were amalgamated with percoll to achieve a final concentration of 15%. This amalgam was then carefully layered onto a step gradient consisting of 23% and 40% percoll. Following this, centrifugation was executed at 24,000 g for 5 min at 4°C (lowered acceleration and with no brakes). The layer on the 15%–23% interphase was meticulously collected, rinsed in isolation buffer, and subjected to centrifugation before being resuspended in synaptosomal buffer (120 mm NaCl, 4.7 mmol/L KCl, 2.2 mm CaCl2, 1.2 mm MgCl2, 25 mmol/L HEPES, 1.2 mmol/L MgSO4, 1.2 mmol/L, KH2PO4, 10 mmol/L glucose). In order to restrict proteolysis, the synaptosomal buffer was supplemented with protease and phosphatases inhibitors cocktail (Thermo scientific, A32961) when stored for Western blotting experiments.

### 2.4 Assessment of NOX activity in isolated synaptosomes by EPR spectroscopy

The assessment of NOX activity involved the detection of NADPH-dependent O_2_^∙−^ generation in synaptosomal fractions through the application of spin-trapping EPR spectroscopy as previously described (Khalifa et al., 2017; Abdel-Rahman et al., 2021) An assortment comprising 10 μL of freshly isolated synaptosomes, 33.25 mM DMPO spin trap, 8.25 mM DETC (to hinder SOD), and a final concentration of 10 mM NADPH was introduced to initiate NOX activity, following which the specimen was placed in a glass capillary and inserted into the EPR cavity of a Benchtop Magnettech MiniScope MS5000 spectrometer (now Bruker Biospin, Berlin) supplied with biotemperature control and computerized data acquisition and analysis capabilities. The DMPO-OH signals stemming from the DMPO-OOH spin adduct were observed continuously for a duration of 7.5 min. Subsequently, the slope was computed over the 7.5-min timeframe utilizing OriginPro software, reflecting the rate of O_2_^∙−^ production attributable to NOX. Normalization of EPR signals was performed based on the protein concentrations of purified synaptosomes as determined utilizing the bradford assay.

### 2.5 Determination of NOX activity in synaptosomes by oroboros high resolution O2k oxygraph

Measurement of NOX respiratory rates was conducted at 37°C utilizing the high-resolution respirometry system Oxygraph-2K (Oroboros Instruments, Innsbruck, Austria) in 2 mL chambers as described elsewhere (Abdel-Rahman et al., 2016; Khalifa et al., 2017). Prior to the commencement of the experiment, calibration at air saturation was performed by allowing the respiration medium, MIR05, to equilibrate with air in the oxygraph chambers and to be stirred at 540–560 rpm for 10–15 min until a stable signal was observed. Synaptosomal protein was introduced into the respiration medium in the chamber. Stimulation of NOX was initiated by the introduction of 200 *μ*M NADPH. Oxygen consumption rates were determined as the negative time derivative of oxygen concentration. The NOX-dependent H_2_O_2_ production rate was measured concurrently with oxygen consumption in the same sample using O2k-Fluorometer module (Oroboros). Measurement of H_2_O_2_ involved the use of horseradish peroxidase (HRP) (1 U per mL) and Amplex UltraRed fluorescent dye (10 *μ*M) with excitation at 525 nm and fluorescence detection at 587 nm. At the peak of NADPH activity leading to oxygen consumption and H_2_O_2_-producing, a specific NOX inhibitor VAS2870 (Chem cruz, sc471103) (100 *μ*M) or GLX351322 (Bio Vision, B2340-5) (10 *μ*M) were added to assess the activity mediated by NOX2 and NOX4 respectively. Data collection and analysis were conducted using the DatLabVR software version 7.4.0.4 (Oroboros Instruments, Innsbruck, Austria), enabling the continuous monitoring and recording of oxygen concentration in the chambers along with the derived oxygen flux over time at rates of 0.5–1 Hz, normalized for the protein concentrations of purified synaptosomes as determined utilizing the bradford assay.

### 2.6 Western blotting

Western blotting analysis on isolated synaptosomes was performed as previously described (Khalifa et al., 2017). Thirty or forty μg of proteins per lane derived from synaptosomes (membrane fraction) isolated from control ASD brains at P15, P30 and P60 were separated using SDS-PAGE and transferred onto PVDF membranes (Serva, 4251401). Following this step, the membranes underwent blocking with either 5% nonfat dry milk (NFDM) or BSA in TBS, supplemented with 0.01% Tween 20 for a duration of 1 h, and subsequently exposed to anti-NOX2 (1:500; in BSA; Invitrogen, MA5-18052), anti-NOX4 (1:250; in BSA; Novus, NB110-58851), anti-NKCC1 (1: 250; in NFDM; Cell signaling, 14581S), anti-KCC2 (1:1000; in BSA; Cell signaling technology, 94725S) and β-actin (1:1000; in NFDM; Invitrogen, MA1-91399) overnight at 4°C. The membranes were then subjected to probing with corresponding anti-rabbit or anti-mouse, HRP conjugated secondary antibodies for 1 h at room temperature (1:2500) and visualized using chemiluminescence reagent and ChemiDoc XRS and gel imaging system (Bio-Rad, Hercules, CA, USA). The bands present were analyzed quantitatively through densitometric assessments utilizing ImageJ analysis software [(National Institutes of Health and the Laboratory for Optical and Computational Instrumentation (LOCI, University of Wisconsin), USA)]. The densities of each band, reflecting individual animals, were normalized to β-actin.

### 2.7 Three chambers test

Three Chambers test was performed as described elsewhere (Baronio et al., 2015). Rats at P60 were individually transferred to the central chamber of a three-chamber maze measuring 120 x 40 x 40(h)cm, where they underwent a 10-minute habituation period. Subsequently, rats were permitted to explore all three chambers for a duration of 10 min, achieved by opening the doors to the side chambers. A novel rat (novel mouse 1), serving as a stranger rat social stimulus, was placed in one side chamber within a wire cage (novel object), while the opposite side chamber contained an empty wire cage to provide a nonsocial stimulus for the sociability test. Following this, the experimental rat was free to navigate through all three chambers for another 10-minute period. For the social novelty test, a different novel rat (novel mouse 2) was introduced into the side chambers, and the experimental rat’s interactions were observed as it approached and sniffed either the familiar rat from the initial phase or the newly introduced novel rat during a 10-minute session. Throughout these experiments, the time spent in each chamber, transitions between chambers, and specific sniffing durations directed towards the empty cage, novel rat, and familiar rat were meticulously recorded. The stranger rats utilized in these tests were carefully matched in terms of age and gender with the experimental rats, and were provided with a 10-minute habituation period on the day preceding the actual testing.

A Sociability Index (SI) was assessed, which is a mathematical formula developed for facilitating the direct comparison of social interactions among different groups. Similarly, an assessment of the inclination towards social novelty was conducted by deriving the Social Novelty Preference Index (SPI). The calculation of both SI and SPI is demonstrated below:

SPI=(Time exploring novel rat 2−Time exploring familiar rat)/(Time exploring novel rat 2+Time exploring familiar rat).

SI=(Time exploring novel rat 1−Time exploring novel object)/(Time exploring novel rat 1+Time exploring novel object).

### 2.8 Open field test

Rats at P60 were situated in the center of a square apparatus, encompassed by vertical walls (60 cm × 60 cm × 40 cm) – referred to as the open-field arena. Rats were engaged in unrestricted exploration of the maze for a duration of 15 min. Their locomotive activities were documented and assessed utilizing the free trial version of video-tracking software–ANY-maze, (Stoelting, Wood Dale, IL, USA). The software utilized the center of the rat’s dorsum as the reference point to ascertain the animal’s position, a practice consistent across all experiments. Analysis involved the delineation of three distinct zones: (1) the region adjacent to the wall (1800 cm^2^); (2) the central area of the arena (900 cm^2^); (3) the area in the corner zones (900 cm^2^). Parameters examined included, total distance traveled, distance traveled in the central zone, average speed, number of immobile episodes and number of defecations.

### 2.9 Statistical analysis

Data are presented as means ± SEM. The normality of the data was assessed by applying Kolmogorov–Smirnov test, Shapiro–Wilk test or D'Agostino’s skewness test in graphpad prism 10.4.1. For data that were normally distributed, statistical significance was assessed by applying the Student’s t-test when variances and sample sizes were equal, or Welch’s t-test when variances or sample sizes were unequal in graphpad prism 10.4.1. The Mann–Whitney U test was employed for groups comparisons, for data that did not follow a normal distribution in graphpad prism 10.4.1.

## 3 Results

### 3.1 Differential synaptic protein expression of KCC2 and NKCC1 in VPA-induced ASD rats throughout development

**FIGURE 2:**
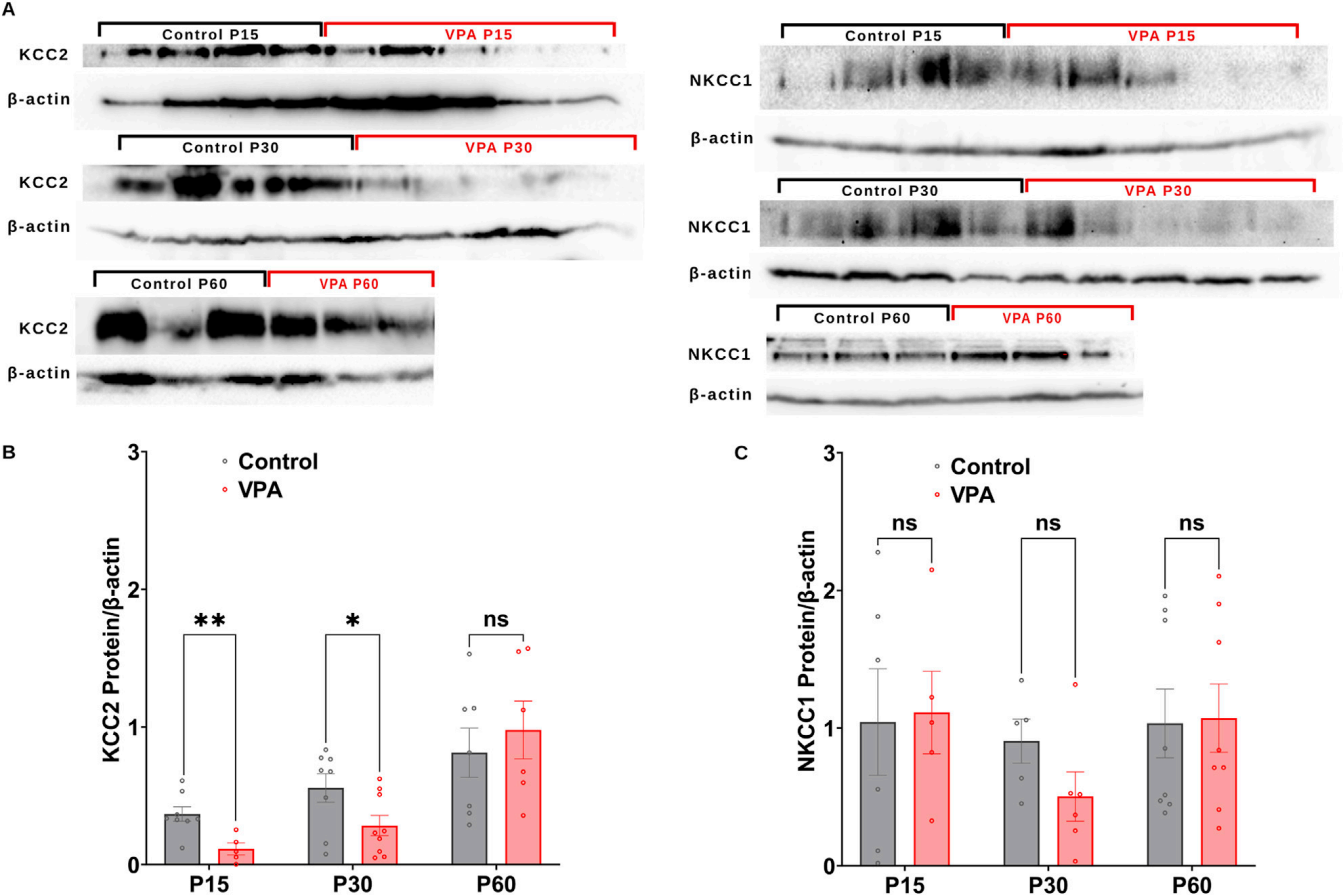
Synaptic protein expression of KCC2and NKCC1 in synaptosomes of control and ASD-induced VPA rats. **(A)** Representative Western blot showing protein expression of KCC2and NKCC1, and β-actin in control and VPA synaptosomes at P15, P30 and P60. Quantification of Western blot data showing developmental profiles of surface protein expression of the KCC2 **(B)**, NKCC1 **(C)**, normalized by β -actin in control and VPA synaptosomes at P15, P30 and P60. Note: The same β-actin loading control was used in Panel **(A)** (NKCC1, P15) and 4A (NOX4, P15). Similarly, the same β-actin loading control was used in Panel **(A)** (NKCC1, P60) and 4A (NOX4, P60).

GABA D/H shift during development is regulated by NKCC1 and KCC2 (Dzhala et al., 2005; Rivera et al., 1999; Yamada et al., 2004). Here, we followed the differential synaptic protein expression of, KCC2 and NKCC1 and in Control-synaptosomes and VPA-synaptosomes at P15, P30, and P60. Western blot analysis revealed that synaptic protein expression of KCC2, is significantly decreased at P15 and P30 in VPA-synaptosomes relative to Control-synaptosomes at corresponding ages [(P15 VPA versus (Vs) P15 control, p = 0.0035; Mean ± SEM, P15 VPA, n = 5: 0.114 ± 0.043, P15 control, n = 8: 0.367 ± 0.052, Figure 2B); (P30 VPA Vs P30 control, p = 0.044; Mean ± SEM, P30 VPA, n = 9: 0.283 ± 0.073, P30 control, n = 8: 0.556 ± 0.103, Figure 2B)]. However, at P60 no significant differences were observed in KCC2 protein expression in VPA-synaptosomes relative to Control-synaptosomes.

Next, we assessed the synaptic protein expression of the chloride importer NKCC1. No significant differences have been observed in NKCC1 protein expression at any developmental stage between VPA-synaptosomes and Control-synaptosomes. Together, these results verify an impaired GABA D/H switch in synapses of VPA-induced ASD rat brains at infantile and juvenile stages (P15 and P30, respectively).

### 3.2 Alterations in NOX-dependent rates of O_2_^∙−^ production, OCR and H_2_O_2_ production rates in synapses of VPA-induced ASD rats throughout development

**FIGURE 3:**
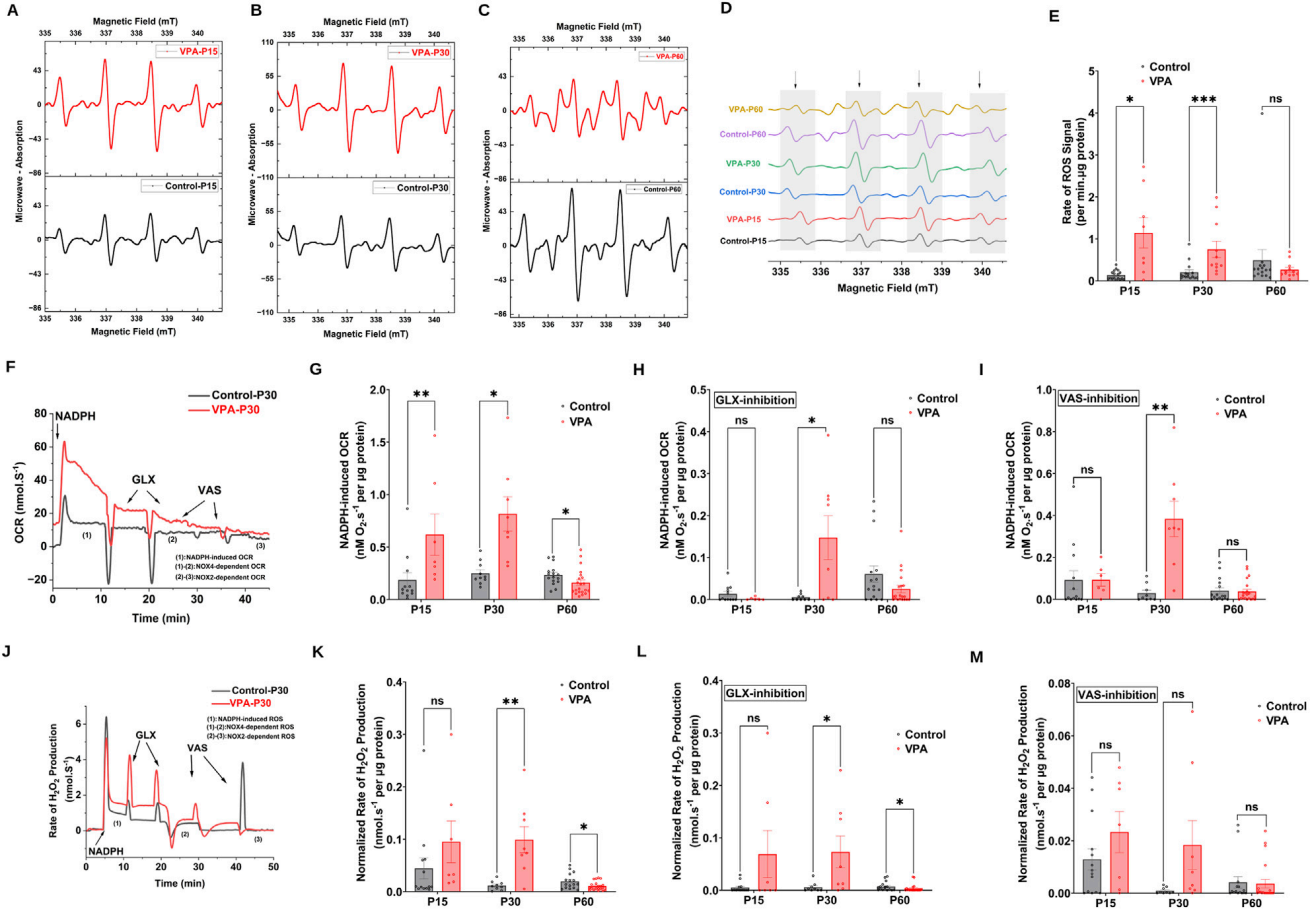
NOX dynamics in synaptosomes of control and ASD-induced VPA rats. **(A–D)** Representatives EPR spectra observed after 7.5 min from NADPH stimulation of NOX in different time points of control and VPA synaptosomes, in the presence of DMPO spin trap. The observed signal is attributable to DMPO-OH spin adduct that arises from superoxide- DMPO as it was completely abolished by exogenous superoxide dismutase. **(E)** Quantifications of age-dependent NADPH-induced rate of superoxide rise signal in control and VPA synaptosomes at P15, P30 and p60. **(F)** Representative traces of NADPH-induced OCR in control and VPA synaptosomes at P30 assessed using high resolution respirometry. NOX activity was induced by adding one dose of NADPH substrate in its reduced form to tightly sealed chamber containing synaptosomes. **(G)** Quantifications of the overall O_2_ fluxes mediated by NOXs in control and VPA synaptosomes at P15, P30 and P60. To confirm the involvement of NOX, we infused the specific NOX4 inhibitor GLX351322 (10 μm) and NOX2 Inhibitor VAS 2870 (10 μm), which indeed remarkably reduced the observed activity. **(H)** Quantifications of the overall O_2_ fluxes mediated by NOX4 isoform in control and VPA synaptosomes at P15, P30 and P60. **(I)** Quantifications of the overall O_2_ fluxes mediated by NOX2 isoform in control and VPA synaptosomes at P15, P30 and P60. **(J)** Representative traces of NOX activity in P30 control and VPA synaptosomes assessed by rate of H_2_O_2_ production using high resolution respirometry combined with HRP/Amplex red fluorescence measurements. NOX activity was induced by adding one dose of NADPH substrate in its reduced form to tightly sealed chamber containing synaptosomes. **(K)** Quantifications of the overall H_2_O_2_ fluxes mediated by NOXs in control and VPA synaptosomes at P15, P30 and P60. **(L)** Quantifications of the overall H_2_O_2_ fluxes mediated by NOX4 in control and VPA synaptosomes at P15, P30 and P60. **(M)** Quantifications of the overall H_2_O_2_ fluxes mediated by NOX2 in control and VPA synaptosomes at P15, P30 and P60.

NOX2 and NOX4, are major sources of ROS at brain synapses, influencing both physiological processes and neurological conditions. Using EPR spin trapping, we measured NADPH-induced O_2_^∙−^ production in synaptosomes from control and VPA-induced ASD rat brains at P15, P30, and P60. Representative EPR traces are presented in Figure 3A, illustrating the enzymatic activity of NOXs within synaptosomes, by monitoring the presence of DMPO-hydroxyl radical adduct, a byproduct derived from the O_2_^∙−^ radical. Our results revealed that synaptosomes isolated from brains of VPA-induced ASD rats at P15 and P30 significantly produce O_2_^∙−^ at higher rates relative to synaptosomes isolated from brains of control rats at comparable ages, [P15 VPA Vs. P15 control, p = 0.0264 Mean ± SEM, P15 VPA, n = 8: 1.141 ± 0.358, P15 control, n = 17: 0.135 ± 0.026, Figures 3D, E); (P30 VPA Vs. P30 control, p = 0.0005; Mean ± SEM, P30 VPA, n = 11: 0.751 ± 0.193, P30 control, n = 15: 0.205 ± 0.057, Figure 3E)]. However, no significant difference in NADPH-induced O_2_^∙−^ production rate in VPA-synaptosomes relative to control-synaptosomes at P60 was observed.

Next, we followed NOX-induced OCR and H_2_O_2_ production rates in synaptosomes isolated from control and VPA rat brains at P15, P30 and P60. Experiments encompassed simultaneous assessments of OCR and H_2_O_2_ generation rates through the utilization of high-resolution respirometry in conjunction with the HRP/Amplex Red fluorescence assay. In Figures 3F, J, we show representative OCR and H_2_O_2_ production rates traces respectively, of Control-synaptosomes and VPA-synaptosomes at P30 as induced by the addition of NADPH. We employed GLX351322 and VAS2870, selective inhibitors for NOX4 and NOX2 [reviewed in: (Fragoso-Morales et al., 2021)], which are members of the NOX family. Notably, through isoform-specific NOX inhibitors, we can start to tease apart the contributions of NOX2 and NOX4 to the VPA-induced cellular and behavioral phenotypes noted herein. Our results showed that activation of endogenous NOX function through NADPH led to elevated levels of oxygen utilization and H_2_O_2_ generation in synaptosomes isolated from the brains of all experimental groups. However, synaptosomes isolated from brains of VPA-induced ASD rats showed significantly higher rates of NADPH–induced OCR at infantile and juvenile stage (P15 and P30 respectively), as well as higher rates of NADPH–induced H_2_O_2_ production at P30 as compared to control synaptosomes at comparable ages; OCR [(P15 VPA Vs. P15 control, p = 0.0072; Mean ± SEM, P15 VPA, n = 7: 0.618 ± 0.196, P15 control, n = 12: 0.185 ± 0.069, Figure 3G); (P30 VPA Vs. P30 control, p = 0.0104; Mean ± SEM, P30 VPA, n = 8: 0.815 ± 0.163, P30 control, n = 10: 0.250 ± 0.034, Figure 3G)]; H_2_O_2_ production rate; (P30 VPA Vs. P30 control, p = 0.0100; Mean ± SEM, P30 VPA, n = 8: 0.011 ± 0.002, P30 control, n = 9: 0.309 ± 0.0285, Figure 3K)]. In contrast, VPA synaptosomes at P60 exhibited a significant reduction in NADPH–induced OCR and H_2_O_2_ production rate compared to control synaptosomes at the same age; OCR (P60 VPA Vs. P60 control, p = 0.0162; Mean ± SEM, P60 VPA, n = 21: 0.160 ± 0.027, P60 control, n = 16: 0.234 ± 0.024, Figure 3G); H_2_O_2_ production rate (P60 VPA Vs. P60 control, 0.0416; Mean ± SEM, P60 VPA, n = 16: 0.01 ± 0.001, P60 control, n = 20: 0.019 ± 0.003, Figure 3K). Next, we determined NOX4 and NOX2-mediated activities in synaptosomes isolated from different experimental groups. Our results revealed that at P30, NOX4-dependent OCR and H_2_O_2_ flux were significantly increased in VPA- synaptosomes relative to control-synaptosomes; OCR (P30 VPA Vs. P30 control, p = 0.0301; Mean ± SEM, P30 VPA, n = 8: 0.147 ± 0.052, P30 control, n = 9: 0.005 ± 0.002, Figure 3H); H_2_O_2_ production rate (P30 VPA Vs. P30 control, p = 0.0215; Mean ± SEM, P30 VPA, n = 8: 0.072 ± 0.03, P30 control, n = 9: 0.005 ± 0.003, Figure 3L). Similarly, at P30, a significant enhancement in NOX2-dependent OCR (P30 VPA Vs. P30 control, p = 0.0038; Mean ± SEM, P30 VPA, n = 8: 0.383 ± 0.084, P30 control, n = 9: 0.029 ± 0.013, Figure 3I); and no significant change in NOX2-dependent H_2_O_2_ flux were observed in VPA-synaptosomes, relative to control-synaptosomes (P30 VPA Vs. P30 control, p = 0.1029; Mean ± SEM, P30 VPA, n = 8: 0.018 ± 0.009, P30 control, n = 9: 9.159 × 10^−4^ ± 4.074 × 10^−4^, Figure 3M). At P15, no significant change in either NOX4-dependent or NOX2-dependent OCR and H_2_O_2_ flux was observed in VPA synaptosomes relative to control synaptosomes. As also shown in Figure 3L, at P60, a significant reduction in NOX4-mediated H_2_O_2_ flux was observed in VPA-synaptosomes compared to control-synaptosomes at the same age (P60 VPA Vs. P60 control, p = 0.0236; Mean ± SEM, P60 VPA, n = 20: 0.025 ± 0.009, P60 control, n = 16: 0.06 ± 0.019, Figure 3L). However, no significant differences were detected between VPA-synaptosomes and control-synaptosomes at P60 in NOX4-mediated OCR, or NOX2-mediated OCR and H_2_O_2_ flux. Taken together, these results suggest that in brains of infantile (P15) and juvenile (P30) VPA-induced ASD rats, activities of synaptic NOXs are increased, while decreased or normalized to control levels in adult stage (P60).

### 3.3 Differential synaptic protein expression of NOX2 and NOX4 subunits in VPA-induced ASD rats throughout development

**FIGURE 4:**
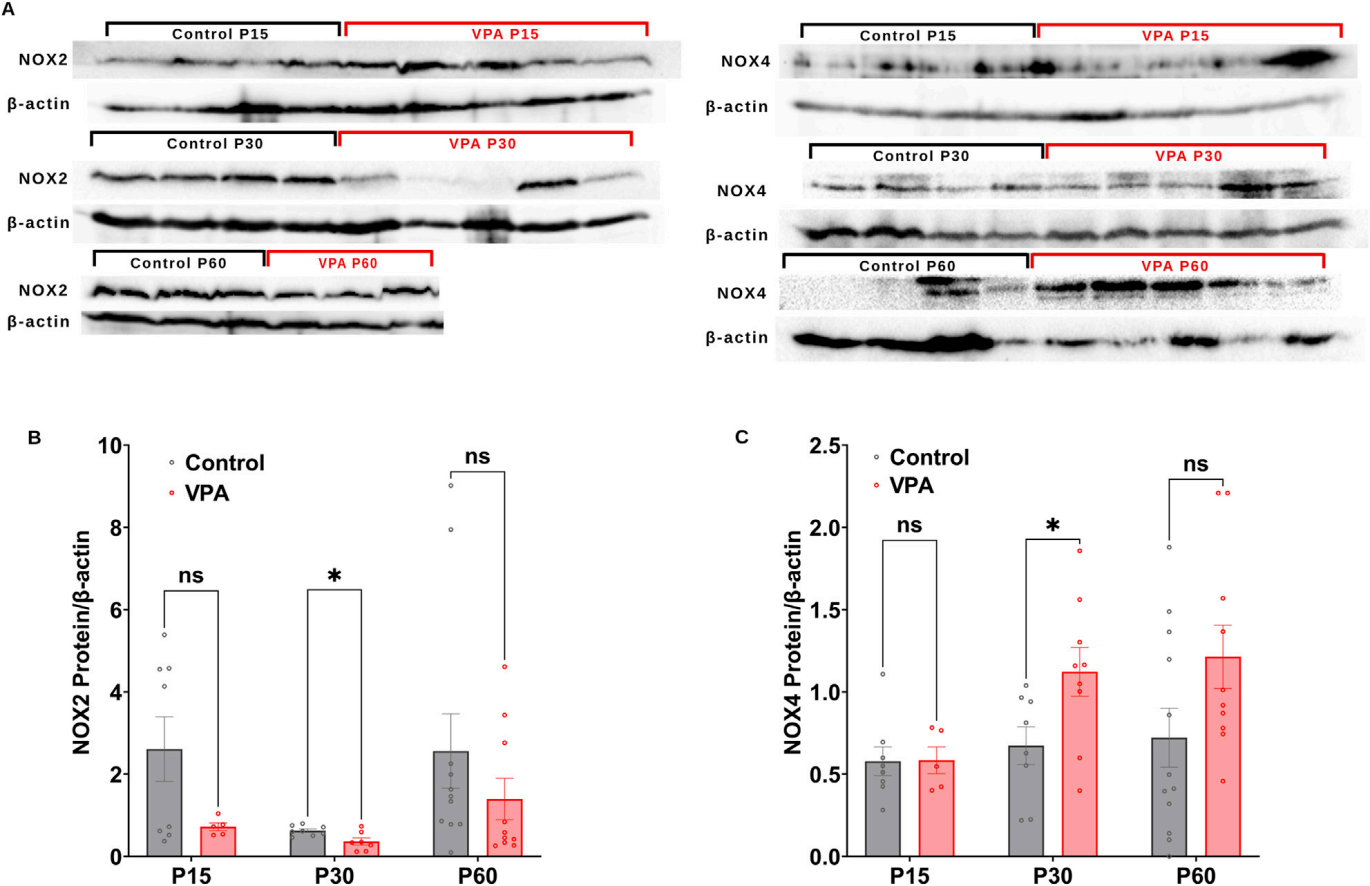
**(A)** Representative Western blot showing protein expression of NOX2, NOX4 subunits and b-actin in control and VPA synaptosomes at P15, P30 and P60. Quantification of Western blot data showing developmental profiles of surface protein expression of the NADPH oxidase catalytic subunits Nox2 **(B)** and Nox4 **(C)** normalized by b-actin in control and VPA synaptosomes at P15, P30 and P60. Values are calculated as relative intensity/µg of loaded protein normalized by β-actin expression and are given as mean ± SEM. Note: The same β-actin loading control was used in Figure 2A (NKCC1, P15) and 4A (NOX4, P15). Similarly, the same β-actin loading control was used in Figure 2A (NKCC1, P60) and 4A (NOX4, P60).

Next, we performed Western blot analysis to investigate differential protein expressions of NOX2 and NOX4, in Control-synaptosomes and VPA-synaptosomes at p15, P30, and p60. At P15 and P60, no significant differences were detected in the relative protein expression of NOX2 in VPA-synaptosomes relative to Control-synaptosomes at corresponding ages. However, at p30, significant reduction in protein expression of NOX2 in VPA-synaptosomes relative to Control-synaptosomes was detected [(P30 VPA Vs. P30 control, p = 0.0239; Mean ± SEM, P30 VPA, n = 7 0.361 ± 0.086, P30 control, n = 8: 0.624 ± 0.042), Figure 4B)]. In contrast, our data showed a significant increase in protein expression of NOX4 at p30 in VPA-synaptosomes relative to Control-synaptosomes (P30 VPA Vs. P30 control, p = 0.032; Mean ± SEM, P30 VPA, n = 9: 1.121 ± 0.148, P30 control, n = 8: 0.672 ± 0.114, Figure 4C). Moreover, although no significant changes in protein expression of NOX4 at p60 in VPA-synaptosomes relative to Control-synaptosomes was detected, a strong trend of enhancement observed (P60 VPA Vs. P60 control, p = 0.076; Mean ± SEM, P60 VPA, n = 12: 1.213 ± 0.193, P60 control, n = 10: 0.721 ± 0.179, Figure 4C). No significant differences in relative protein expression of NOX4 in VPA-synaptosomes compared to Control-synaptosomes at P15 was observed.

### 3.4 Effect of prenatal exposure to shikonin on synaptic protein expression of KCC2 and NKCC1, in VPA-induced ASD rat

**FIGURE 5:**
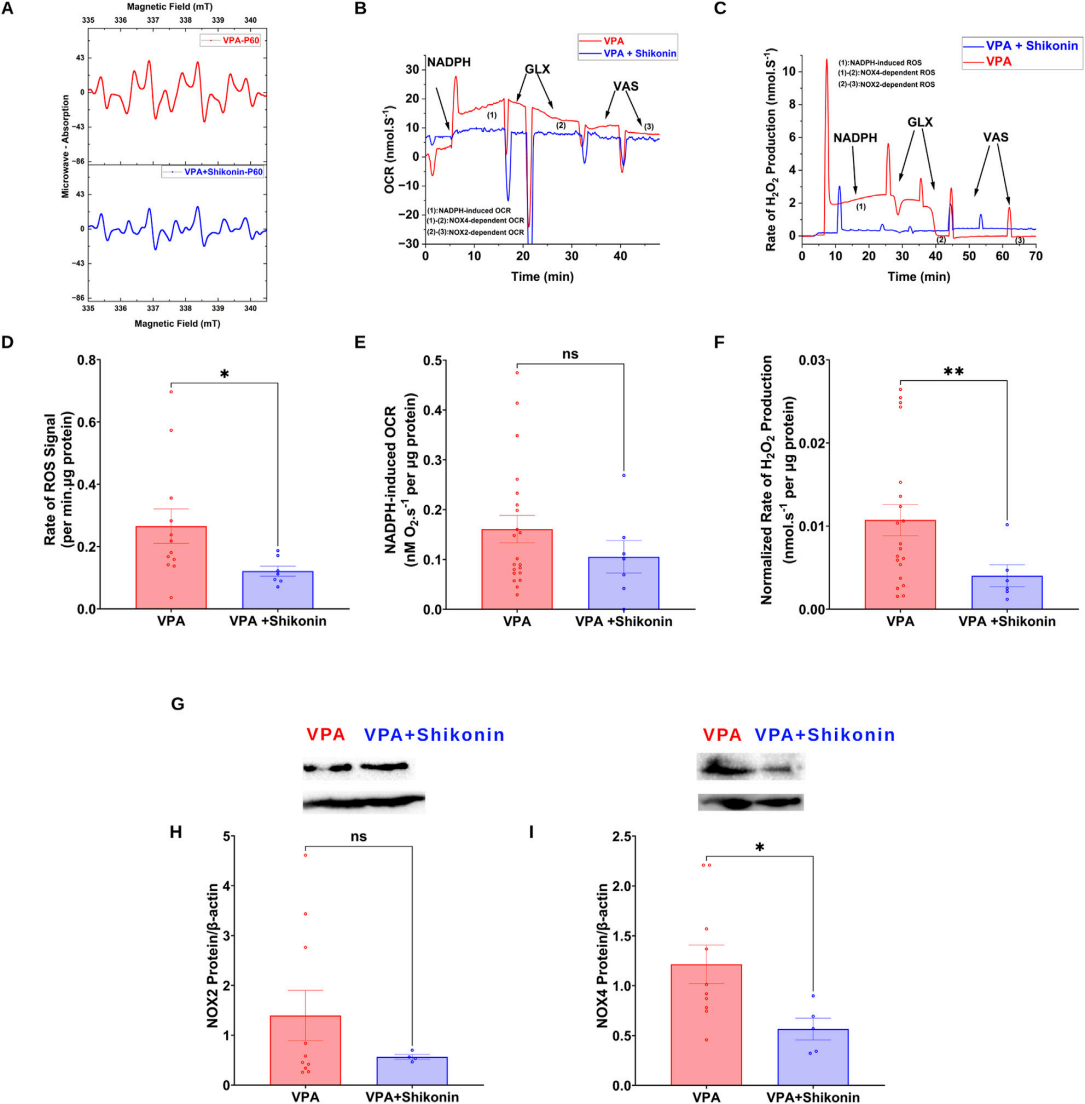
Effect of prenatal exposure to shikonin on dynamics of synaptic NOXs in VPA-induced ASD rat **(A)** Representatives EPR spectra observed after 15 min from NADPH stimulation of NOX in VPA and VPA + Shikonin synaptosomes, in the presence of DMPO spin trap. **(B)** Representative traces of NADPH-induced OCR in VPA and VPA + Shikonin synaptosomes assessed using high resolution respirometry. **(C)** Representative traces of NOX activity in VPA and VPA + Shikonin synaptosomes assessed by rate of H_2_O_2_ production using high resolution respirometry combined with HRP/Amplex red fluorescence measurements. **(D)** Quantifications of NOX-dependent O_2_∙− in VPA, VPA + Shikonin treated groups’ synaptosomes. **(E)** Quantifications of the overall O_2_ fluxes mediated by NOX in VPA, VPA + Shikonin synaptosomes. **(F)** Quantifications of the overall H_2_O_2_ fluxes mediated by NOX in VPA, VPA + Shikonin synaptosomes. **(G) (A)** Representative Western blot showing protein expression of NOX2, NOX4 subunits and b-actin in VPA and VPA + Shikonin synaptosomes **(H)** Quantification of surface protein expression of NOX2 normalized by β-actin in VPA, VPA + Shikonin synaptosomes. **(I)** Quantification of surface protein expression of NOX4 normalized by β-actin in VPA, VPA + Shikonin synaptosomes. Note: The same β-actin loading control was used in Panel **(G)** (NOX2) and 6A (KCC2).

**FIGURE 6:**
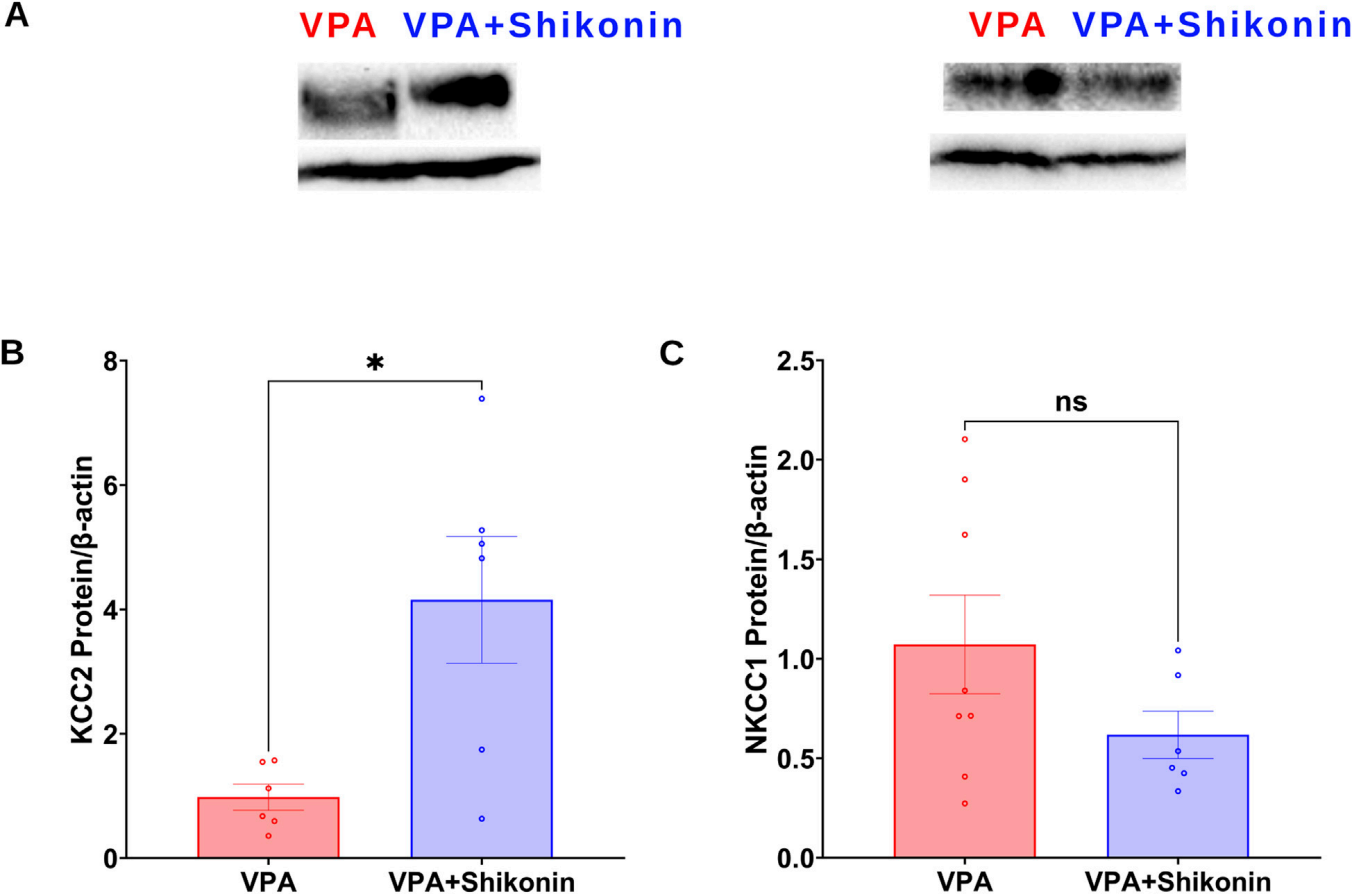
Effect of prenatal exposure to shikonin on synaptic protein expression of KCC2and NKCC1 in VPA-induced ASD rat. **(A)** Representative Western blot showing protein expression of KCC2, NKCC1 and β-actin in VPA, VPA + Shikonin. Quantification of surface protein expression of KCC2 **(B)** and NKCC1 **(C)** and in VPA, VPA + Shikonin at P60. Values are calculated as relative intensity/µg of loaded protein normalized by b-actin expression and are given as mean ± SEM.

Shikonin, a naphthoquinone from *Lithospermum erythrorhizon* roots, reduces oxidative stress by inhibiting NOX2 and NOX4 enzymes activities and expression in doxorubicin-induced cardiotoxicity (Kazumura et al., 2016), and sepsis-induced acute kidney injury (Peng et al., 2022) animal models respectively. First, we assessed the effect of prenatal exposure to shikonin on synaptic NOXs activities in VPA-induced ASD rats (Figures 5A–C). Our results showed a significant inhibition of NOXs-mediated O_2_^∙−^ production rates in shikonin + VPA synaptosomes (VPA Vs. VPA+ Shikonin, p = 0.0266; Mean ± SEM, VPA, n = 12: 0.265 ± 0.055, VPA+ Shikonin, n = 7: 0.121 ± 0.016, Figure 5D); as well as H_2_O_2_ generation rates in shikonin + VPA synaptosomes (VPA Vs. VPA + Shikonin, p = 0.0080; Mean ± SEM, VPA, n = 18: 0.01 ± 0.001, VPA+ Shikonin, n = 7: 0.004 ± 0.001, Figure 5F). No significant effect of prenatal exposure to shikonin on NOX-mediated OCR in Shikonin + VPA synaptosomes; Figure 5E. Next, we explored the effect of prenatal exposure to shikonin on synaptic protein expression of NOX4 and NOX2 in VPA-synaptosomes. Interestingly, shikonin significantly reduced NOX4 protein expression in VPA-synaptosomes (VPA Vs. VPA + Shikonin, p = 0.0120; Mean ± SEM, VPA, n = 10:1.213 ± 0.193, VPA+ Shikonin, n = 5: 0.564 ± 0.108, Figure 5I). However, no significant effect of prenatal exposure to shikonin on synaptic protein expression of NOX2 was detected. Finally, we assessed the effect of *in utero* modulation of NOX-induced ROS, by shikonin on synaptic protein expression of KCC2 and NKCC1 in VPA-synaptosomes. Interestingly, shikonin enhanced protein expression of KCC2 in VPA + Shikonin-synaptosome relative to VPA-synaptosomes (VPA Vs. VPA+ Shikonin, p = 0.0256; Mean ± SEM, VPA, n = 6: 0.978 ± 0.209, VPA+ Shikonin, n = 6: 4.153 ± 1.019, Figure 6B). However, no significant effect of shikonin on synaptic protein expression of NKCC1 in VPA + Shikonin-synaptosome relative to VPA synaptosomes was observed.

### 3.5 Impact of prenatal exposure to shikonin on abnormal behaviors in VPA-induced ASD rats

**FIGURE 7:**
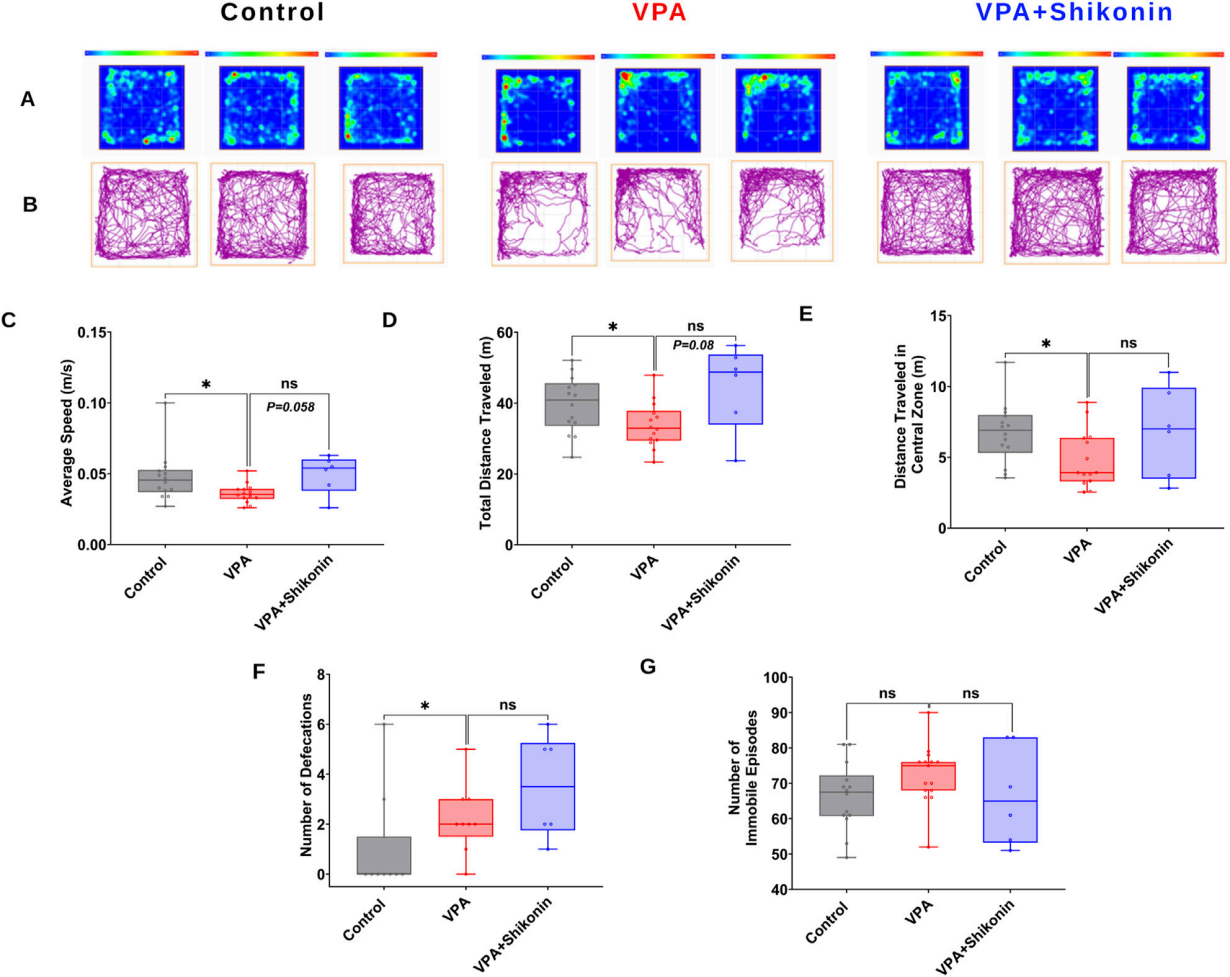
The impact of prenatal exposure to shikonin on behavioral alterations in the open-field test in VPA-induced ASD rats. Representatives heat maps **(A)** and track plots **(B)** for control, VPA and shikonin + VPA exposed rats in the open field test. Quantification of the average speed of rats **(C)** the total distance traveled by rats **(D)**, distance traveled in the central zone **(E)**, number of defecations **(F)** and number of immobile episodes recorded for each rat **(G)** in control, VPA and shikonin + VPA exposed rats.

**FIGURE 8:**
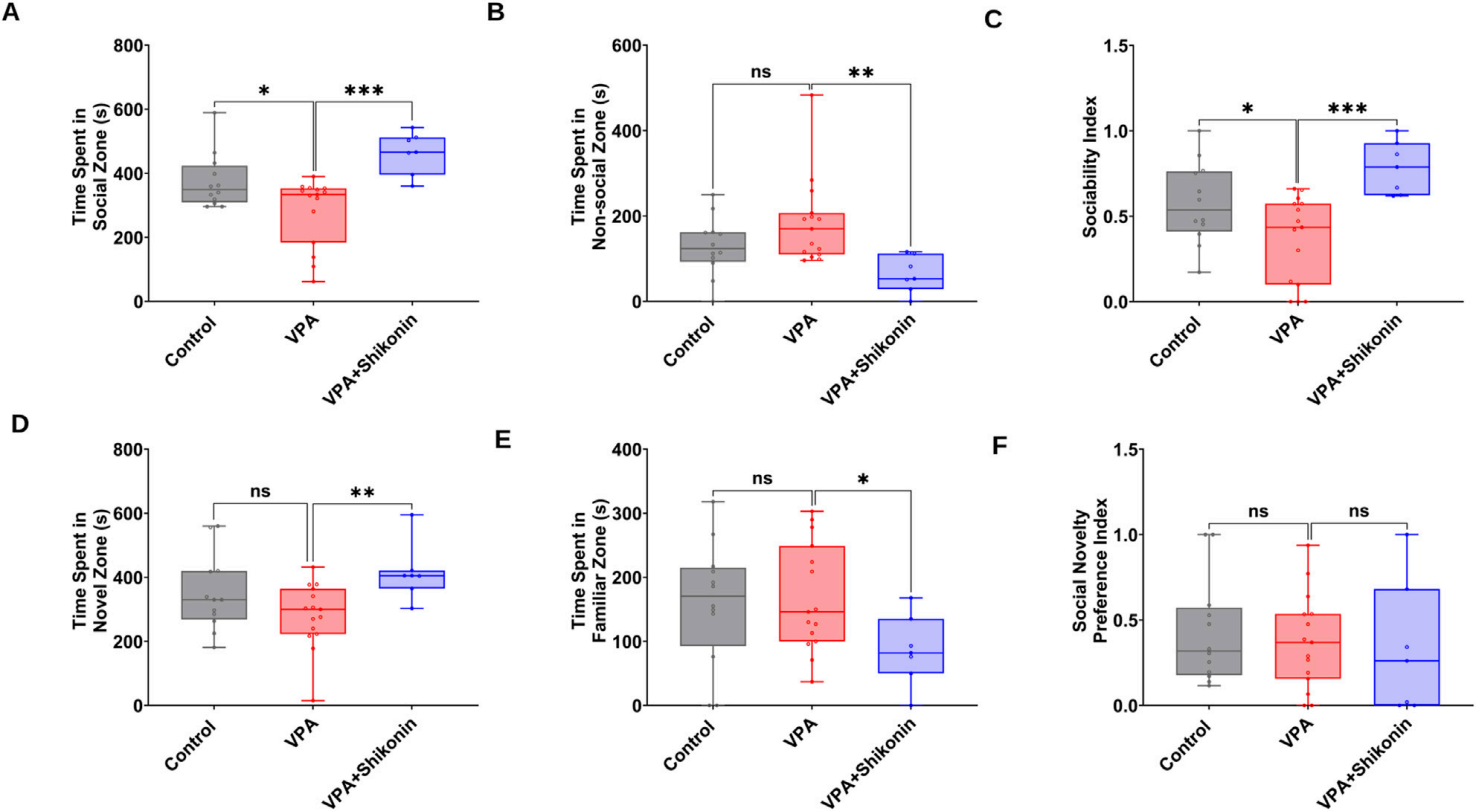
The impact of prenatal exposure to shikonin on behavioral alterations in the three chambers social interaction test in VPA-induced ASD rats. Quantification of the time spent in the social zone **(A)** The time spent in the non-social zone **(B)** Sociability index **(C)** calculated from the time spent exploring the social (stranger rat1) versus non-social (Object) stimulus) in the three chambers test. Quantification of the time spent in novel zone **(D)**, The time spent in familiar zone **(E)** and Social novelty preference index **(F)** derived from the time exploring the novel (Stranger rat 2) versus familiar (Stranger rat 1) stimulus in the three chambers test.

First, we verified behavioral deficits in animals prenatally subjected to VPA by performing open- field and three chambers social interaction tests. Compared to control rats, the average speed (Control rats Vs. VPA rats, p = 0.0338; Mean ± SEM, Control, n = 14: 0.047 ± 0.004, VPA, n = 14: 0.036 ± 0.001, Figures 7A–C), the total distance travelled and the distance travelled in the central zone by VPA-induced ASD rats were significantly lower, suggesting inferior exploratory activity Figures 7D, E. Additionally, relative to control animals, VPA-induced ASD rats exhibited significantly higher numbers of defecations (Control rats Vs. VPA rats, p = 0.0448; Mean ± SEM, Control, n = 14: 66.285 ± 2.521, VPA, n = 15: 72.4 ± 2.172, Figure 7F) as well as a strong trend of increased number of immobile episodes (Figure 7G). In the three chambers sociability test, VPA-induced ASD rats spent significantly less time in the social zone (novel rat 1 chamber), than control rats (Control rats Vs. VPA rats, p = 0.023; Mean ± SEM, Control, n = 12: 374.5 ± 24.815, VPA, n = 15: 283.4 ± 27.163, Figure 8A). No significant difference in the time spent in the novel object chamber (non-social zone) was observed between VPA-induced ASD rats and control rats. The SI of VPA subjected rats was significantly lower than the SI of control rats (Control rats Vs. VPA rats, p = 0.0352; Mean ± SEM, Control, n = 12: 0.576 ± 0.068, VPA, n = 15: 0.3635 ± 0.06545, Figure 8C), indicative of impaired sociability in VPA-induced ASD rats. Regarding the social novelty preference, no significant difference in the time spent in the novel zone (novel rat 2 chamber) between VPA-induced ASD rats and control rats was observed. Similarly, no significant difference in the time spent in the familiar zone (familiar rat chamber) was observed between VPA-induced ASD rats and control rats. The SPI did not differ to any significant extent in VPA-induced ASD rats and control rats, suggesting no change in social novelty. Next, we investigated the effect of prenatal exposure to shikonin on ameliorating behavioral deficits in VPA-induced ASD rats. Although the differences in the average speed activity and the total distance travelled by VPA-induced ASD rats relative to VPA-induced ASD rats prenatally exposed to shikonin did not reach statistical significance (p > 0.05), the data indicate a trend toward enhanced exploratory activity by prenatal shikonin exposure. (Average speed: VPA rats Vs. VPA+ Shikonin rats, p = 0.0580; Mean ± SEM, VPA, n = 14: 0.036 ± 0.001, VPA+ Shikonin, n = 6: 0.049 ± 0.005, Figure 7C); (Total distance travelled: VPA rats Vs. VPA+ Shikonin rats, p = 0.0817; Mean ± SEM, VPA, n = 14: 33.82 ± 1.714, VPA+ Shikonin, n = 6: 44.641 ± 4.929, Figure 7D). Additionally, Prenatal exposure to shikonin did not elicit any significant changes in distance travelled in the central zone, number of defecations and number of immobile episodes in VPA-induced ASD rats. Regarding the sociability test, VPA-induced ASD rats prenatally exposed to shikonin spent significantly more time in the social zone (Novel rat 1 chamber) compared to VPA-induced ASD rats, (VPA rats Vs. VPA+ Shikonin rats, p = 0.0001; Mean ± SEM, VPA, n = 15: 283.4 ± 27.163, VPA+ Shikonin, n = 7,463.428 ± 24.644, Figure 8A). Moreover, prenatal exposure to shikonin significantly decreased the time spent in non-social zone (Novel object chamber) by VPA-induced ASD rats (VPA rats Vs. VPA+ Shikonin rats, p = 0.0008; Mean ± SEM, VPA, n = 15: 184.666 ± 26.158, VPA+ Shikonin, n = 7: 63.285 ± 16.138, Figure 8B). In addition, VPA-induced ASD rats prenatally exposed to shikonin displayed significantly higher SI compared to VPA-induced ASD not exposed to shikonin (VPA rats Vs. VPA+ Shikonin rats, p = 0.0001; Mean ± SEM, VPA, n = 15: 0.3635 ± 0.1, VPA+ Shikonin, n = 7: 0.784 ± 0.057, Figure 8C). These findings suggest that prenatal exposure to shikonin ameliorated deficits in sociability in VPA-induced ASD rats. Concerning the social novelty preference test, although prenatal exposure to shikonin significantly enhanced the time spent by VPA rats in the novel zone (novel rat 2 chamber) (VPA rats Vs. VPA+ Shikonin rats, p = 0.0081; Mean ± SEM, VPA, n = 15: 281.333 ± 26.189, VPA+ Shikonin, n = 7: 414 ± 33.733, Figure 8D), and significantly decreased the time spent in familiar (Familiar rat chamber) by VPA-induced ASD rats (VPA rats Vs. VPA+ Shikonin rats, p = 0.0142; Mean ± SEM, VPA, n = 15: 168.2 ± 21.813, VPA+ Shikonin, n = 7: 86.285 ± 20.691, Figure 8E), SPI was not affected by shikonin. Taken together, these findings suggest that relative to control rats, VPA-induced ASD rats displayed impaired exploratory activity, and deficits in sociability, with no change in social novelty preference. Prenatal exposure to shikonin ameliorated behavioral deficits in VPA-induced ASD rats.

## 4 Discussion

Within the adult brain, GABA serves as the main inhibitory neurotransmitter, whereas in immature neurons, GABA exerts a depolarizing and excitatory effect (Ben‐Ari et al., 1989). GABAARs-mediated neurotransmission possesses a distinct characteristic in that its function can be simply altered through modifications of the ionic driving force. In mature neurons, a diminished intracellular chloride concentration leads to an equilibrium potential for chloride ions (ECl), that is to some extent hyperpolarized relative to the neuronal resting membrane potential (V_rest_). Indeed, elevated chloride concentration in immature neurons, reverses the polarity of GABAARs-mediated currents from hyperpolarizing to depolarizing. Development-associated D/H switch transpires over an extended temporal scale and gives rise to hyperpolarizing effects of GABA within the fully developed brain (Hartmann and Nothwang, 2022). Major players in the regulation of the D/H shift encompass the secondary active membrane transport proteins NKCC1 and KCC2. In immature neurons, enhanced expression of NKCC1 causes accumulation of chloride, which is subsequently extruded upon opening of GABAARs, leading to membrane depolarization. Upon neuronal maturation, the increased expression of the chloride importer KCC2 results in attenuation of intracellular chloride concentration that facilitates chloride influx via GABAARs, resulting in hyperpolarization. Accordingly, the existence of altered expression of NKCC1 and KCC2 levels within the mature brain is commonly regarded as a hallmark of an immature excitatory GABAergic profile (Ben‐Ari et al., 1989; Dzhala et al., 2005; Rivera et al., 1999; Yamada et al., 2004).

In the current study, we identified that KCC2 protein levels are significantly lower in synaptosomes from VPA-induced ASD rats at P15 and P30 but return to normal levels by P60 (Figure 2B). In tune with our current results, previous studies showed a reduction of KCC2 protein expression in the hippocampus of VPA rats at P15 (Tyzio et al., 2014) and P30 (Haratizadeh et al., 2023; Tyzio et al., 2014) and the hippocampus of Fmr1-KO mice, model of Fragile X syndrome (FRX) (rare type of ASD) at P15 and P30 (Tyzio et al., 2014). Downregulation of KCC2 has also been detected in primary neurons derived from murine Lgdel+/−, which serve as a model of DiGeorge Syndrome, a rare ASD resulting from the deletion of a segment on chromosome 22 (Amin et al., 2017). The lack of change in the synaptic protein expression of KCC2 in VPA synaptosome observed at P60, is however not in agreement with the study by Savardi et al., showing dysregulation of KCC2 in cortex of adult VPA mice as well as in prefrontal cortices of adult human subjects with non-genetic variants of ASD (Savardi et al., 2020). This discrepancy in KCC2 protein expression at P60 could stem from differences in types of samples used (Synaptosomes isolated from whole brain of VPA rats in the current study Vs cortex of adult VPA mice and/or prefrontal cortices of adult human in the study by Savardi et al. (Savardi et al., 2020). NKCC1 protein levels are unchanged in synaptosomes from VPA-induced ASD rats at all ages studied (Figure 2C). These results agree with previous studies showing no significant differences in NKCC1 expression in VPA-induced ASD rats (Li et al., 2017), in cortex of adult VPA mice and prefrontal cortex of postmortem ASD brains (Savardi et al., 2020). Given that we found significant downregulation of synaptic protein expression of KCC2, and no significant change in synaptic protein expression of NKCC1 in VPA rats at P15 and p30, this could indeed hint at aberrant development-associated D/H transition of GABA at brain synapses of VPA-induced ASD rats at infantile and juvenile stages. To the best of our knowledge, this is the first report that addresses in detail, developmental stages–dependent changes in synaptic protein expression of KCC2, NKCC1 in brains of VPA induced ASD rats throughout infantile to juvenile and adult stages.

The persistence of GABA-mediated depolarization induces a long-lasting activation of NMDARs, and subsequently excessive calcium influx and excitotoxic neuronal cell damage (reviewed in: Armada-Moreira et al., 2020). NMDAR-mediated excitotoxicity has been demonstrated as a crucial mechanism in the development of oxidative stress and the consequent cellular damage within ASD brains (Essa et al., 2013; Nisar et al., 2022). Previous reports have indicated that NOXs are the primary contributors to the production of O_2_^∙−^ in response to sustained stimulation of NMDARs (Brennan et al., 2009; Han et al., 2016; Kovac et al., 2014; Wang and Swanson, 2020). Within brain synapses, NOXs have become progressively acknowledged as major sources of oxidative stress. We have previously reported synaptic localization of NOX2 and NOX4 isoforms in the brains of control C57BL6 male and female mice, and their pivotal roles in ROS generation within synapses (Abdel-Rahman et al., 2016; Khalifa et al., 2017). We propose a new vicious cycle contributing to ASD behavioral deficits, involving a disrupted GABA D/H shift during development, glutamate excitotoxicity, and NOX overactivation at synapses. This cycle leads to sustained synaptic ROS production. We followed synaptic NOXs activities and protein expressions in brains of VPA-induced ASD rats at P15, P30 and P60. The present study used EPR spin trapping spectroscopy to assess rates of production of O_2_∙−, the primary product of NOX enzymes, in synaptosomes isolated from brains of VPA induced ASD rats and control rats at P15, P30 and P60 (Figures 3A–C). The capability of the EPR method lies in its capacity to simultaneously detect, identify, and semi-quantify different ROS in real-time. We also determined NOX-mediated OCR and H_2_O_2_ production rates using the Oroboros high-resolution O2k Station (Figures 3D–K). Synaptic activities of NOXs are boosted in VPA-induced ASD rats at infantile (P15) and juvenile (P30) stages; whereas have been reduced or normalized to control levels in adult stage (P60) (Figure 3F). In tune with this, one recent study showed that, in a rat model of prenatal maternal immune activation by lipopolysaccharide (LPS), the activity of NOXs is decreased in the whole brain homogenate of adolescent rats (P54) (Cieślik et al., 2023). We also observed that NOX2 synaptic protein expression decreases and NOX4 synaptic protein expression increases at P30 (Figures 4A,B, respectively), suggesting a switch in NOX2 and NOX4 isoforms protein expression in VPA- synaptosomes at P30. Of note, the observed increase in NOX activity at P30 might be due to NOX4 upregulation. To the best of our knowledge, this is the first report that addresses in detail, developmental stages–dependent changes in synaptic NOXs expression and NOXs activities in brains of VPA induced ASD rats throughout infantile to juvenile and adult stages.

Shikonin has been shown to hinder the generation of O_2_^∙−^ by NOX2 in neutrophils (Kazumura et al., 2016). Furthermore, Shikonin diminished NOX2 activity, reversed the increase in NOX2 subunit expression, and mitigated cardiotoxicity in mice treated with doxorubicin (Tuo et al., 2024). Additionally, through the regulation of NOX4 expression, shikonin mitigated ROS production and improves sepsis-induced acute kidney injury in rats (Peng et al., 2022). To answer the question “Can it be that modulation of NOXs could prevent impairment in the development-associated D/H shift of synaptic GABA and subsequent behavioral deficits in VPA-induced ASD rats?”, we followed the effect of prenatal exposure to shikonin on the synaptic protein expression of KCC2 and NKCC1, as well as behavioral deficits in VPA-induced ASD rats. Our data indicates that prenatal exposure to shikonin significantly reduces NOX-mediated ROS production and NOX4 protein levels in VPA-induced ASD rats. We also observed that shikonin mitigates dysregulation in GABA D/H switch by modulating synaptic protein expression of KCC2 in VPA-induced ASD rats (Figure 6B).

There is ample evidence for a role for oxidative stress in altering the excitatory/inhibitory shift of GABAA receptors in pathological conditions. For example, in hepatic encephalopathy, oxidative stress decreases KCC2 expression leading to impaired GABA inhibition in the substantia nigra pars reticulata, and motor dysfunction. Antioxidant treatment restored KCC2 expression and ameliorated oxidative stress symptoms (Bai et al., 2018). Reactive environmental toxicants (e.g., bisphenol A, lead (Pb^2+^) also induce oxidative stress and inflammation, leading to altered KCC2, NKCC1 expressions and impaired synaptic and mitochondrial function (Neuwirth et al., 2018). In addition, oxidative stress induces signaling pathways such as the oxidative stress–responsive kinase OSR1, which through no-lysine kinase (WNK)- SPS1-related proline/alanine rich kinase (SPAK) signaling, phosphorylates and modulates NKCC1 and KCC2 activities (reviewed in: (Huang et al., 2019; Beltrán González et al., 2020)). OSR1 is mainly recognized for the regulation of chloride co-transporters during osmotic stress (Watanabe and Fukuda, 2015), yet its activation by oxidative stress suggests redox-sensitive mode of post-translational control. Supporting this, a phosphoproteomics study revealed that OSR1 was a novel substrate of Ataxia-telangiectasia mutated (ATM kinase) in the context of hydrogen peroxide-induced oxidative stress (Kozlov et al., 2016). Enhanced ATM levels have been observed in two autism models, Rett syndrome and prenatal VPA exposure, exhibiting delayed GABAergic switch (Pizzamiglio et al., 2021). Interestingly, inhibition of ATM can reinstate the expression of KCC2, which rescues abnormal GABAergic signaling, as well as autism-like behaviors, in mice. (reviewed in: (Abruzzo et al., 2021)). Taken together, these findings imply that oxidative stress has the ability to replicate genetic or chemical insults that lead to a dysregulation of KCC2 expression or activity and, consequentially, of GABAergic signaling.

Since we have demonstrated enhanced NOX-mediated ROS production in synapses of infantile and Juvenile VPA-induced ASD rats, it would be interesting to see in future studies whether ROS produced by NOXs modulate the synaptic activity of GABAARs as well as WNK/SPAK/OSR1-mediated phospho-regulation and chloride transport activity of NKCC1 and KCC2 in synapses of VPA-induced ASD rats.

The VPA-induced ASD animal model displays behavioral impairments akin to those seen in individuals with autism. In tune with previous studies, our results revealed impaired exploratory activity (Gąssowska-Dobrowolska et al., 2020; Schneider and Przewłocki, 2005) in rats exposed to VPA in the open field test. Our findings are also in accordance with previous studies demonstrating impaired sociability (Baronio et al., 2015; Gąssowska-Dobrowolska et al., 2020; Scheggi et al., 2020; Vörös et al., 2023), as indicated by lower SI (Figures 8A–C). However, social novelty preference is not affected in VPA exposed rats, (Figure 8F), in agreement with previous studies (Gąssowska-Dobrowolska et al., 2020; Dai et al., 2018). A, Prenatal exposure to shikonin ameliorated deficits in sociability in VPA-induced ASD rats. Additionally, a tendency toward increased exploratory behavior was observed in VPA-animals prenatally exposed to shikonin, though it did not reach statistical significance. The impact of prenatal exposure to shikonin on alleviating abnormal behaviors in VPA-induced ASD rats in the present study, is in tune with the results of a previous report by Le Belle et al., (Le Belle et al., 2014), showing that prenatal inhibition of NOX with apocynin could rescue the excessive grooming behavior, at P20 in pups subjected to MIA. However, the same study (Le Belle et al., 2014) also showed that the early vocalization deficit in MIR-exposed pups were not improved by NOX inhibition by apocynin. Taken together, findings of the current study suggest that shikonin, by modulating synaptic NOX-ROS prevents disruptions in the development-related GABA D/H switch and the resulting behavioral deficits in VPA-induced ASD rats.

## 5 Conclusion

The current investigation offers a novel perspective on the dynamics of synaptic NOXs during development in VPA-induced ASD rats. Nevertheless, the study did have some limitations. First, the effect of prenatal exposure to shikonin on synaptic protein expression of GABA D/H shift machinery and synaptic NOXs dynamics is only followed at P60, when most changes in VPA-induced ASD rats were either diminished or have been recovered to normal levels at this stage.

Here, we reported for the first time abnormalities in the synaptic NOXs activity in the brains of VPA-induced ASD rats, spanning from the infantile to juvenile and adult stages. We show for the first time that synaptic NOXs activities peaked in the brains of VPA-induced ASD rats at the infantile and juvenile stages, but this was not sustained through the adult stage. Similarly, alterations in synaptic protein expression of GABA D/H switch machinery in VPA-induced ASD rats were only observed at the infantile and juvenile stages, while they faded away by the adult stage. This can be attributed to the fact that the persistence of GABA-mediated depolarization at the juvenile stage in brains of VPA-induced ASD rats, may induce sustained glutamate excitotoxicity and NOX overactivation, resulting in irreversible neuronal loss that is manifested in the adult brains of VPA-induced ASD rats. Put differently, initial oxidative damage might have induced permanent changes to neural circuitry. Prenatal exposure to shikonin modulated synaptic protein expression of NOX4 & KCC2 and ameliorated behavioral deficits in adult VPA-induced ASD rats. It would be interesting to detect the dose-dependent impact of prenatal exposure to shikonin on these parameters in VPA-induced ASD rats at this specific stage. Additionally, it is intriguing to investigate the impact of prenatal exposure to shikonin on the synaptic activity of GABAARs as well as the chloride transport activity of KCC2 and NKCC1 in VPA-induced ASD rats.

## Data Availability

The raw data supporting the conclusions of this article will be made available by the authors upon request, without undue reservation.

## Supplementary material

SUPPLEMENTARY FIGURE S1 Western blots scans. Scanned Western blots; **(A)** and **(B)** = Figures 2A,B (P15); **(C)** and **(D)** = Figures 4A,B (P15); **(E)** and **(F)**= Figures 2A,B (P30); **(G)** and **(H)**= Figures 4A,B (P30); **(I)** and **(J)** = Figures 2A,B (P60); **(K)** and **(L)** = Figures 4A,B (P60); **(M)** and **(O)** = Figures 2A,C (P15); **(N)** and **(O)** = Figures 4A,C (P15); **(P)** and **(Q)** = Figures 2A,C (p30); **(R)** and **(S)** = Figures 4A,C (P30); **(T)** and **(U)** = Figures 2A,C (P60); **(V)** and **(W)** = Figures 4A,C (P60). Note: The same β-actin loading control was used in Figure 2A (NKCC1, P15) and Figure 4A (NOX4, P15). Similarly, the same β-actin loading control was used in Figure 2A (NKCC1, P60) and Figure 4A (NOX4, P60).

SUPPLEMENTARY FIGURE S2 Western blots scans. Scanned Western blots; **(A)** and **(D)** = Figures 6A, C; **(B)** and **(G)** =Figures 5G,H; **(C)** and **(F)** = Figures 5G,I; **(E)** and **(G)** = Figures 6A,B. Note: The same β-actin loading control was used in Figure 5G (NOX2) and Figure 6A (KCC2).
