# Supplementary figures and images for: Prenatal modulation of NADPH-oxidase reverses the deranged GABA switch and rescues behavioral deficits in valproate ASD rat model
Source: Front Pharmacol. 2025 May 30;16:1571008. doi: 10.3389/fphar.2025.1571008 (PMC12162577; doi:10.3389/fphar.2025.1571008)

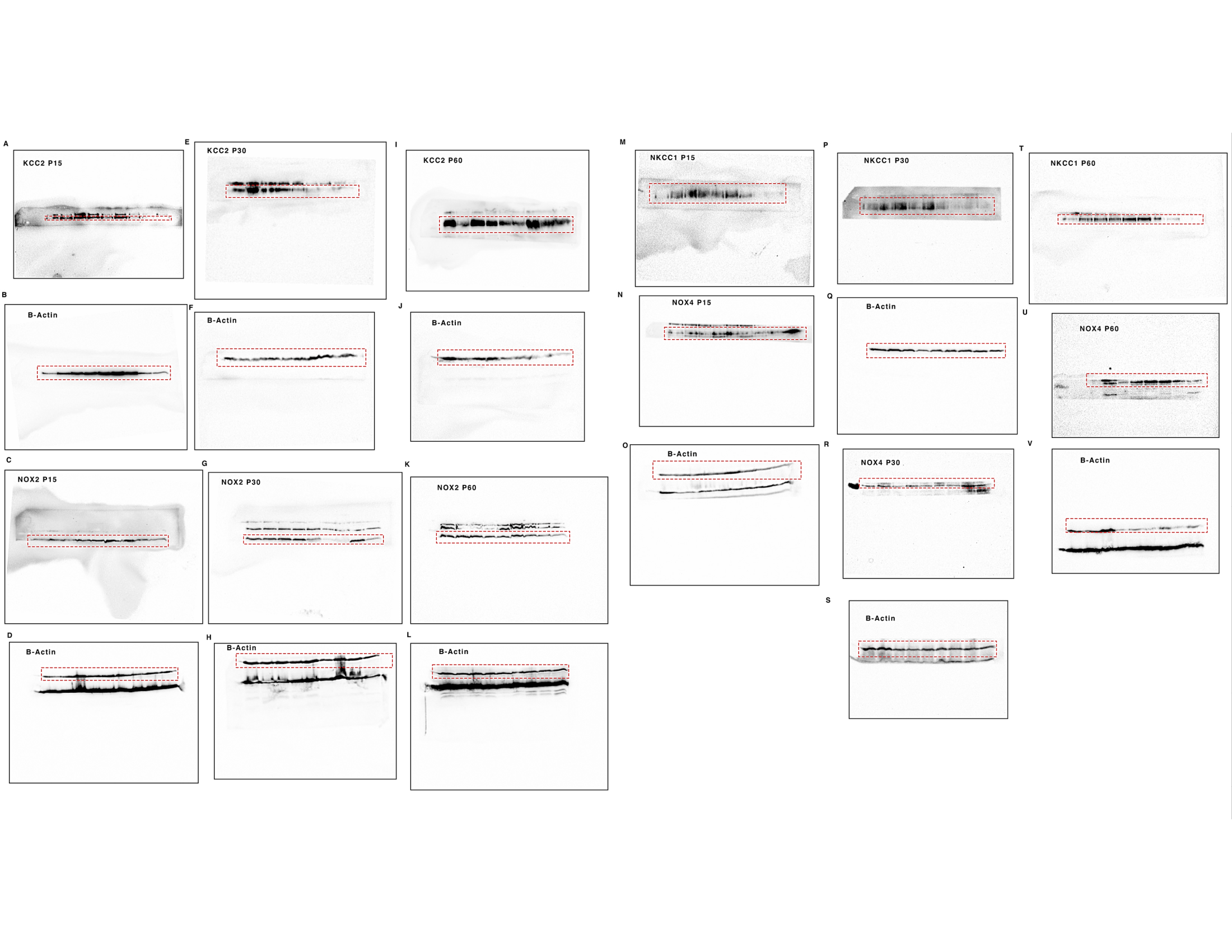

Supplement: Supplementary file 1 [file Image1.tiff]

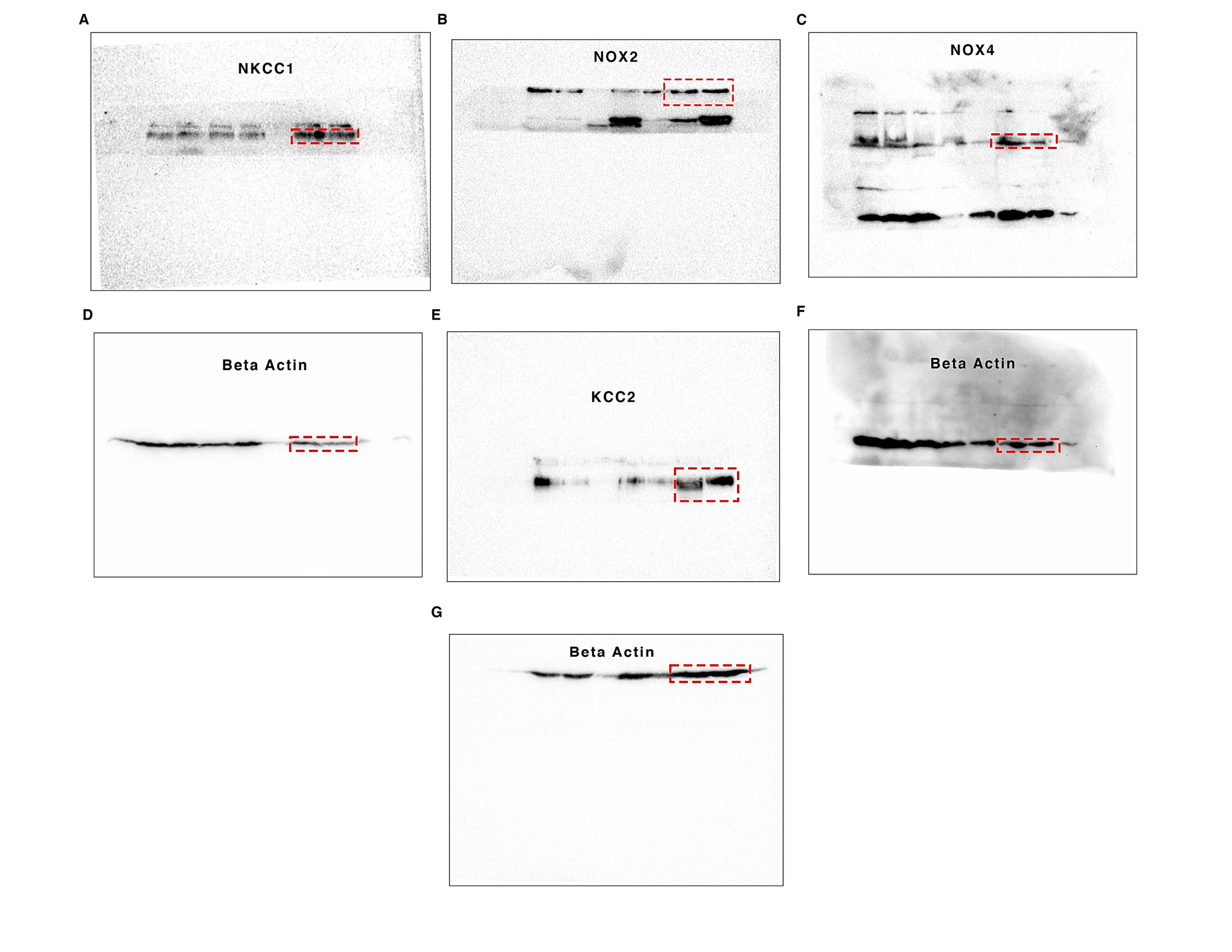

Supplement: Supplementary file 2 [file Image2.tiff]
